# Vascular endothelial growth factor-C secretion is increased by advanced glycation end-products: possible implication in ocular neovascularization

**Published:** 2012-10-11

**Authors:** Alessandra Puddu, Roberta Sanguineti, Arianna Durante, Massimo Nicolò, Giorgio L. Viviani

**Affiliations:** 1 Department of Internal Medicine and Medical Specialties, Viale Benedetto, Genova, Italy; 2 Department of Neuroscience, Ophthalmology and Genetics, Viale Benedetto, Genova, Italy

## Abstract

**Purpose:**

Neovascularization is a common complication of many degenerative and vascular diseases of the retina. Advanced glycation end-products (AGEs) have a pathologic role in the development of retinal neovascularization, mainly for their ability in upregulating vascular endothelial growth factor-A (VEGF-A) secretion. The aim of this study was to investigate whether AGEs are able to modulate the secretion of VEGF-C, another angiogenic factor that increases the effect of VEGF-A.

**Methods:**

A human retinal pigment epithelial cell line (ARPE-19) and human endothelial vascular cell line (HECV) cells were cultured for 24 h in presence of AGEs, and then mRNA expression of VEGF-C was analyzed with reverse transcription–polymerase chain reaction (RT–PCR). To verify whether AGEs-induced VEGF secretion is mediated by RAGE (Receptor for AGEs), RAGE expression was depleted using the small interfering RNA method. To investigate whether VEGF-A is involved in upregulating VEGF-C secretion, the cells were cultured for 24 h in the presence of bevacizumab, a monoclonal antibody against VEGF-A, alone or in combination with AGEs. VEGF-A and VEGF-C levels in the supernatants of the treated cells were evaluated with enzyme-linked immunosorbent assay.

**Results:**

Exposure to AGEs significantly increased VEGF-C gene expression in ARPE-19 cells. AGEs-induced VEGF-C secretion was upregulated in retinal pigment epithelium (RPE) and endothelial cells. Downregulation of RAGE expression decreased VEGF-A secretion in cell models, and increased VEGF-C secretion in ARPE-19 cells. Adding bevacizumab to the culture medium upregulated constitutive VEGF-C secretion but did not affect AGEs-induced VEGF-C secretion.

**Conclusions:**

These findings suggest that AGEs take part in the onset of retinal neovascularization, not only by modulating VEGF-A but also by increasing VEGF-C secretion. In addition, our results suggest that VEGF-C may compensate for treatments that reduce VEGF-A.

## Introduction

Neovascularization, i.e., abnormal formation of new vessels from preexisting capillaries in the retina, is a common complication of many degenerative and vascular diseases of the retina. It is well established that vascular endothelial growth factor-A (VEGF-A) plays a central role in several degenerative and vascular diseases of the retina and choroid, such as diabetic retinopathy (DR) and age-related macular degeneration (AMD), resulting in a significant visual loss among patients with diabetes mellitus [1-3].

The retinal pigment epithelium (RPE), a monolayer of highly specialized cells located between the retinal photoreceptors and the choroidal vasculature [4,5], contributes significantly to the constitutive retinal VEGF-A expression [6]. Furthermore, RPE cells secrete more VEGF-A toward the basolateral or choroid side [7], possibly facilitating its action on choriocapillaris. In addition, overexpression of VEGF-A in RPE cells of the retina might be a responsible factor in the development of choroidal neovascularization (CNV) in vivo [8,9]. Recent studies suggest that VEGF-C, another member of the VEGF family produced by RPE cells, may also play a role in retinal neovascularization. Indeed, VEGF-C shows structural analogies related to VEGF-A [10] and, like VEGF-A, induces mitogenesis and migration of endothelial cells, and promotes capillary-like formation by choroidal endothelial cells in vitro [11,12]. Furthermore, overexpression of VEGF-C in DR protects vascular endothelial cells from apoptosis and consequently promotes choroidal neoangiogenesis [12].

Advanced glycation end-products (AGEs) are a heterogeneous group of molecules that physiologically accumulate during aging and at a faster rate in diabetic individuals than in healthy subjects [13,14]. AGEs are important mediators of vascular diabetic complications, including retinopathy [13,14]. A pathological role of AGEs has been recognized in age-related macular degeneration and DR [14-19]. Many of the adverse effects of AGEs are the result of several factors, including the formation of the protein cross-link that alters the structure and function of the extracellular matrix, the generation of oxidative stress, and the interaction with specific receptors [20,21]. Intracellular effects of AGEs result in oxidative stress and in proinflammatory gene activation, and are mainly mediated by RAGE, the only receptor for AGEs that has a role in signal transduction [21,22].

Several studies have shown that AGEs modulate the function of RPE and endothelial cells affecting the expression of angiogenic factors [23,24]. AGEs increase VEGF-A secretion in RPE and endothelial cells, while the regulation of VEGF-C secretion by AGEs in these cells has not yet been studied [16,25]. In this work, we investigate the possible modulation of VEGF-C secretion in RPE and endothelial cells exposed to a glycated environment.

## Methods

### Advanced glycation end-products preparation

Glycated serum (GS) was prepared by adding 50 mmol/l ribose to heat-inactivated (56 °C for one hour) fetal bovine serum (FBS; Cambrex Bio Science, Walkersville, MD) as described in Viviani et al. [26]. Aliquots of FBS were processed in the same way but without ribose solution (non-glycated serum [NGS]) and used for standard medium preparation. Pentosidine (PENT) content was evaluated as a measure of protein glycation, using the combined reverse-phase ion-exchange chromatographic assay [27]. In the experimental media (Dulbecco’s modified Eagle medium [DMEM]/F12 containing 10% GS), the concentration of PENT ranged between 250 and 260 nmol/l (corresponding to the plasma level found in diabetic patients).

### Cell culture and experimental conditions

Human retinal pigment epithelial cell line (ARPE-19) cells from passages 22 to 26 (American Type Culture Collection, Manassas, VA) were grown in a 1-to-1 ratio of DMEM/F12 (Cambrex Bio Science) supplemented with 10% FBS, 2 mmol/l-glutamine (Sigma-Aldrich, Milan, Italy), and antibiotics (100 U/ml penicillin G and 100 μg/ml streptomycin sulfate; Sigma-Aldrich). Cells were maintained under similar culture conditions during all the experiments. The human endothelial cell line HECV (Cell Bank and Culture in GMP, IST Genoa, Italy) was grown in DMEM supplemented with 10% FBS, 2 mmol/l-glutamine (Sigma-Aldrich). Cells were maintained at 37 °C in a humidified 5% CO_2_-95% air incubator. The medium was replaced every 2 days. Cells were grown to confluence, removed with trypsin-EDTA (Sigma-Aldrich, Milan, Italy), and then seeded in multiwell plates for all experiments.

Before each experiment, confluent cells were washed twice with PBS (Cambrex Bio Science), and fresh medium was added. Cells were cultured in the following media: standard medium (CTR) and medium in which NGS was replaced with GS (AGEs). All experiments were performed after 24 h of culture in the above described conditions.

### Cell viability

To evaluate cell proliferation, ARPE-19 and HECV cells were plated in a 96-well plate (2×10^4^ cells/well) and cultured for 24 h as described above. Viable cells were identified using the Cell Titer 96 Aqueous One Solution Cell Proliferation Assay (Promega, Milan, Italy) according to the manufacturer’s instructions. Briefly, it is a colorimetric method that determines the number of viable cells via MTS tetrazolium reduction into a colored formazan product directly proportional to the number of living cells in culture [26].

### Vascular endothelial growth factor secretion

ARPE-19 and HECV cells were cultured for 24 h in standard conditions or in the presence of AGEs. A set of experiments was performed on cells in which RAGE expression was inhibited by RNA interference. Another set of experiments was performed in the presence of 0.25 mg/ml of the humanized anti-VEGF-A monoclonal antibody bevacizumab (BE; Genentech, Inc.). To quantify VEGF-A and VEGF-C secretion, the conditioned media were collected and stored at –80 °C until the assay was performed. Cells were then washed twice with PBS and lysed in radioimmunoprecipitation assay (RIPA) buffer. The lysates were stored at –80 °C. The lysate protein content was determined with the BCA Protein Assay Kit (Pierce, Rockford, MD) according to the manufacturer’s instructions. VEGF-A and VEGF-C secretions were assessed with enzyme-linked immunosorbent assay (ELISA; Bender MedSystem, Vienna, Austria). VEGF-A and VEGF-C concentrations were calculated from the standards curve and normalized to the total protein concentration of the respective lysate.

### RNA interference

ARPE-19 and HECV cells were grown to 60%–80% confluence and then transfected. RAGE gene silencing was performed using a mix of three RAGE small interfering RNAs (siRAGE) or a negative control with an irrelevant sequence (siControl; Santa Cruz Biotechnology Inc., Santa Cruz, CA) with the appropriate transfection reagent diluted in the transfection medium (Santa Cruz Biotechnology Inc.). Transfection mixtures were left on the cells for 5 h, and then a medium containing two times the normal serum was added. After 18 h incubation, the medium was replaced with fresh growth medium. The cells were treated with AGEs 24 h after the transfection, and RAGE expression was determined by immunoblotting 48 h post-transfection.

### Reverse transcription–polymer chain reaction

Total RNA was extracted from ARPE-19 and HECV cells with the RNeasy kit (Qiagen s.r.l., Milan, Italy) according to the manufacturer’s instruction. The RNA concentrations were determined spectrophotometrically, and equal quantities of total RNA from different samples were used. One microgram of RNA was reverse-transcripted to cDNA using the GoScript Reverse Transcription System (Promega) and then amplified with PCR. The primers for human VEGF-A and for glyceraldehyde-3-phosphate dehydrogenase (GAPDH) were designed according to their mRNA sequences from the GenBank. GAPDH was used as the internal control. The oligonucleotide primers used for the amplification of human VEGF-A cDNA were 5′-ATG GCA GAA GGA GGG CAG CAT-3′ (sense) and 5′-TTG GTG AGG TTT GAT CCG CAT CAT-3′ (antisense). The resultant PCR product was 255 bp. The oligonucleotide primers used for amplifying human GAPDH cDNA were 5′-TGA AGG TCG GAG TCA ACG GAT TTG GT-3′ (sense) and 5′-CAT GTG GGC CAT GAG GTC CAC CAC-3′ (antisense). The resultant PCR product was 558 bp. Primers for VEGF-C amplification were from SABiosciences (Qiagen). The resultant PCR product was 180 bp. The cDNA was amplified using the PCR Master Mix (Promega). Each cycle consisted of 30s at 94 °C, 60s at 62 °C for amplifying VEGF-A and GAPDH cDNA, 60 °C for VEGF-C cDNA, and 60s at 72 °C. All the reactions finished with an extension step of 10 min at 72 °C. All the samples were amplified in a linear amplification range established using a serial cDNA dilution and varying the number of cycles (35 cycles for GAPDH and VEGF-C; 40 cycles for VEGF-A). PCR products were electrophoresed onto a 1.5% agarose gel containing ethidium bromide and visualized under ultraviolet (UV) light. The relative intensities of the bands were quantified with densitometric analysis.

### Immunoblotting analysis

ARPE-19 and HECV cells were lysed in RIPA buffer (50 mmol/l Tris HCl pH 7.5, 150 mmol/l NaCl, 1% NP-40, 0.1% sodium dodecyl sulfate, supplemented with protease and phosphatase inhibitor cocktails), and the protein concentrations were determined using the BCA Protein Assay Kit. Thirty micrograms of total cell proteins were separated on 12% sodium dodecyl sulfate–PAGE and transferred onto nitrocellulose (GE Healthcare UK Ltd, Buckinghamshire, England). Filters were blocked in 5% non-fat dried milk and incubated overnight at 4 °C with primary antibodies specific to: RAGE (Chemicon International, Temecula, CA), and β-Actin (Santa Cruz Biotechnology Inc.). Secondary specific horseradish-peroxidase linked antibodies (GE Healthcare UK Ltd) were added and left for 1 h at room temperature. Bound antibodies were detected using the enhanced chemiluminescence lighting system (ECL Plus, GE Healthcare UK Ltd), according to the manufacturer’s instructions. Bands of interest were quantified with densitometry.

### Statistical analysis

All statistical analyses were performed using the GraphPad Prism 4.0 software (GraphPad Software, San Diego, CA). Data were expressed as the mean±SEM and then analyzed with *t* tests or one-way ANOVA (ANOVA) and using Bonferroni’s method as the post-test. A p value <0.05 was considered statistically significant. The results are representative of at least three experiments.

## Results

ARPE-19 and HECV cells cultured for 24 h in media containing AGEs did not display any differences in cell morphology and viability compared to control cells (data not shown).

### Advanced glycation end-products increase vascular endothelial growth factor-C expression

**Figure 1:**
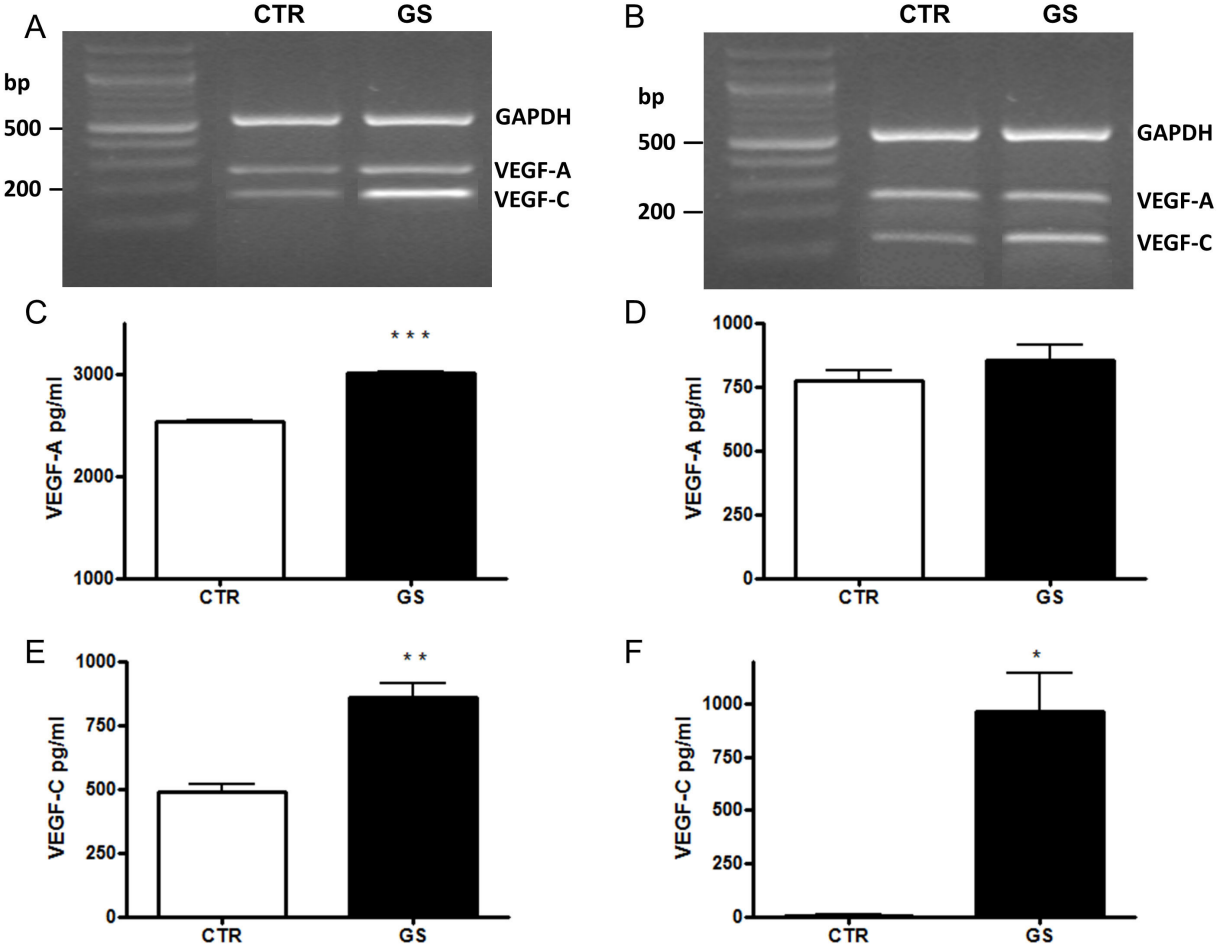
Reverse transcription–polymerase chain reaction (RT–PCR) analysis of VEGF-A and VEGF-C. RT–PCR analysis of VEGF-A and VEGF-C mRNA expression, respectively, in ARPE-19 **A** and HECV **B** cells cultured 24 h in standard conditions (CTR) or in the presence of AGEs (GS). GAPDH was used as the internal control. VEGF-A and VEGF-C secretion in ARPE-19 **C**, **E:** and HECV **D**, **F:** cells, respectively, after 24 h culture in CTR or in presence of AGEs (GS). The conditioned media were collected, and ELISA was performed. Concentrations of VEGFs were calculated from standards curves and normalized to total protein. The results are representative of at least three experiments (mean±SEM). *p<0.05; **p<0.01; ***p<0.001.

First we investigated the expression and secretion of VEGF-A. We found that AGEs increased the expression and secretion of VEGF-A in ARPE-19 cells, but not in HECV (Figure 1A-D). Then we investigated whether AGEs affect VEGF-C mRNA expression in ARPE-19 cells. The RT–PCR data clearly showed that AGEs upregulated VEGF-C mRNA (Figure 1A).

Since CNV develops as a result of pathophysiological changes occurring in the RPE layer as well as in choroidal tissues, the expression of VEGF-C was also investigated in the endothelial cell line HECV cultured for 24 h with AGEs. RT–PCR analysis showed that AGEs slightly upregulated VEGF-C mRNA (Figure 1B).

### Advanced glycation end-products increase vascular endothelial growth factor-C secretion

To verify whether the increase in VEGF-C mRNA was coupled to a rise in VEGF-C secretion, the conditioned medium was collected after exposure to AGEs for 24 h, and VEGF-C concentration was determined with ELISA. The results showed that treatment of ARPE-19 and HECV cells with AGEs significantly increased VEGF-C secretion compared to standard culture (Figure 1E,F).

**Figure 2:**
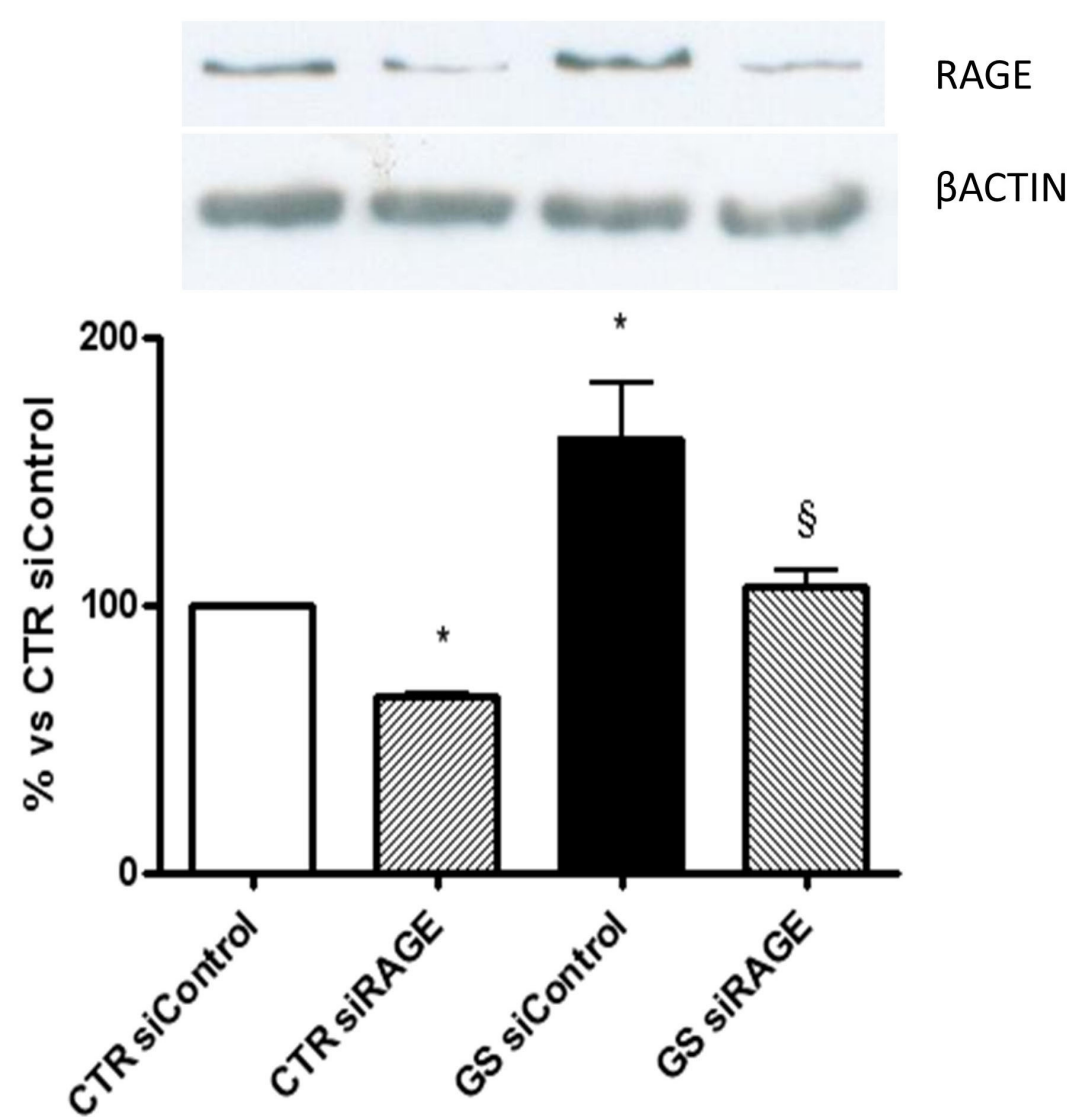
Expression of receptor for AGEs (RAGE). Expression of RAGE in ARPE-19 cells transfected with control siRNA or siRNA of RAGE (siRAGE) following 24 h of culture in standard conditions (CTR) or in the presence of AGEs (GS). Cells were lysed, and lysates were processed for immunoblot. Cells were lysed, and lysates were processed for immunoblot. Bands were visualized with an ECL detection system, and intensity was quantified with densitometric analysis using NIH Image software and expressed as fold induction relative to β-actin. Data shown are representative of at least three experiments (mean±SEM). *p<0.05 versus CTR siControl; ^§^p<0.05 versus GS siControl. Comparable results were obtained in HECV (data not shown).

**Figure 3:**
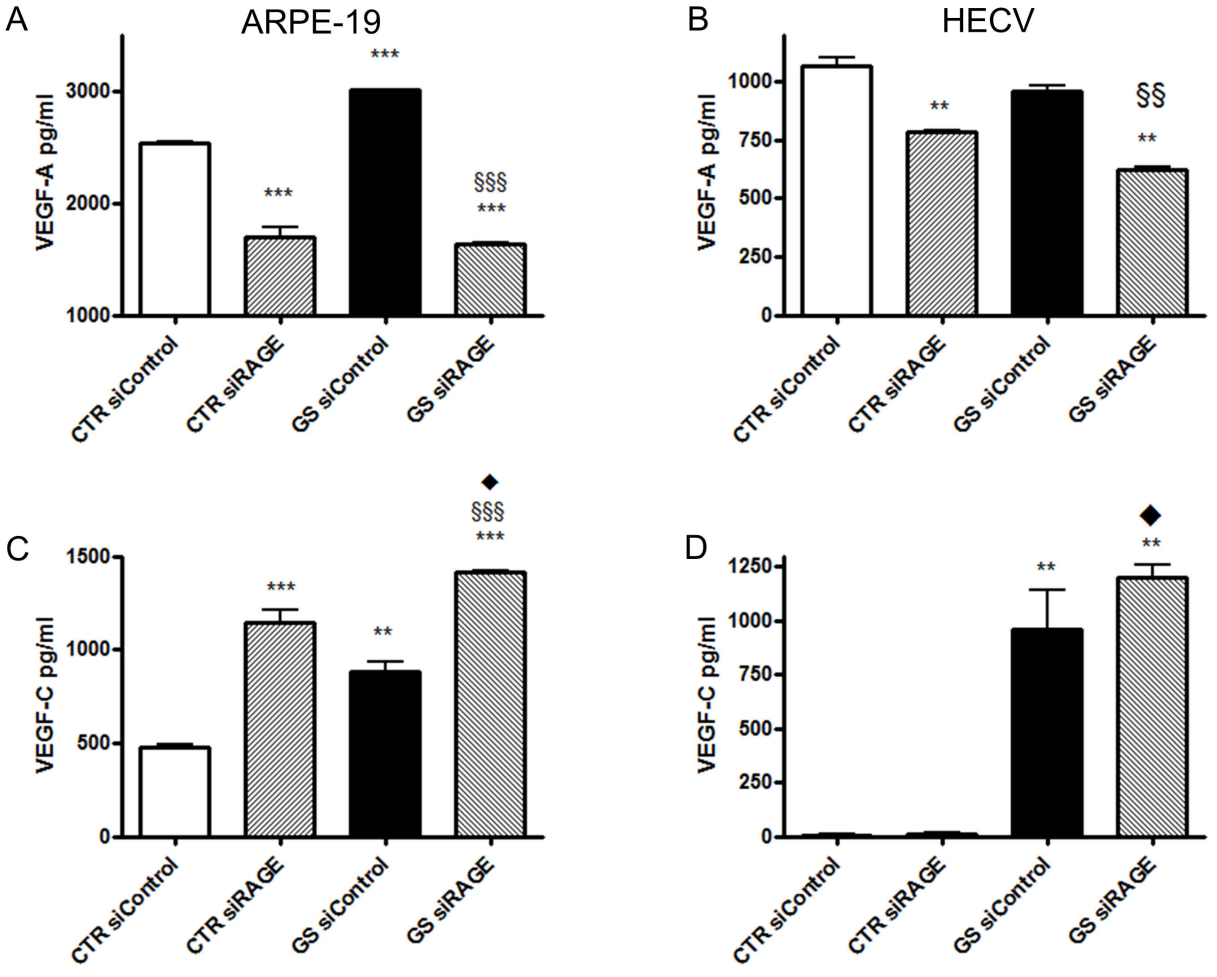
Secretion of vascular endothelial growth factor (VEGF)-A and –C. VEGF-A and VEGF-C secretion in ARPE-19 (**A**, **C** and HECV **B**, **D** cells, respectively, transfected with a scrambled siRNA sequence (siControl) or siRNA of RAGE (siRAGE) following 24 h of culture in standard conditions (CTR) or in the presence of AGEs (GS).The conditioned media were collected, and ELISA was performed. Concentrations of VEGFs were calculated from standards curves and normalized to total protein. The results are representative of at least three experiments (mean±SEM). **p<0.01 and ***p<0.001 versus CTR siControl*;* ^§§^ p<0.01 and ^§§§^ p<0.001 versus GS siControl*;* ^♦^p<0.05 versus CTR siRAGE.

Intracellular effects of AGEs are mediated by RAGE. Binding of AGEs to RAGE results in a positive feedback signal that increases RAGE expression [22]. To investigate whether AGEs-induced VEGFs secretion is mediated by RAGE, we downregulated RAGE expression through specific RAGE siRNAs. We obtained a 40% reduction in RAGE expression in cells transfected with siRAGE compared with those transfected with siControl (Figure 2). No difference in VEGF secretion between untreated cells and siControl cells was observed (data not shown). Interestingly in ARPE-19 cells cultured in standard conditions and in which RAGE expression was downregulated (CTR siRAGE cells) VEGF-A secretion decreased compared to the same cells transfected with a scrambled siRNA sequence (CTR siControl cells; Figure 3A); on the contrary, VEGF-C secretion increased (Figure 3C). When ARPE-19 cells in which RAGE expression was downregulated were exposed to AGEs (GS siRAGE cells), the secretion of VEGF-A was comparable to that of CTR siRAGE cells, while the release of VEGF-C was significantly increased.

The same experimental setting was performed with HECV (Figure 3B,D). Downregulation of RAGE expression decreased VEGF-A secretion, without affecting either constitutive or AGEs-induced VEGF-C release.

**Figure 4:**
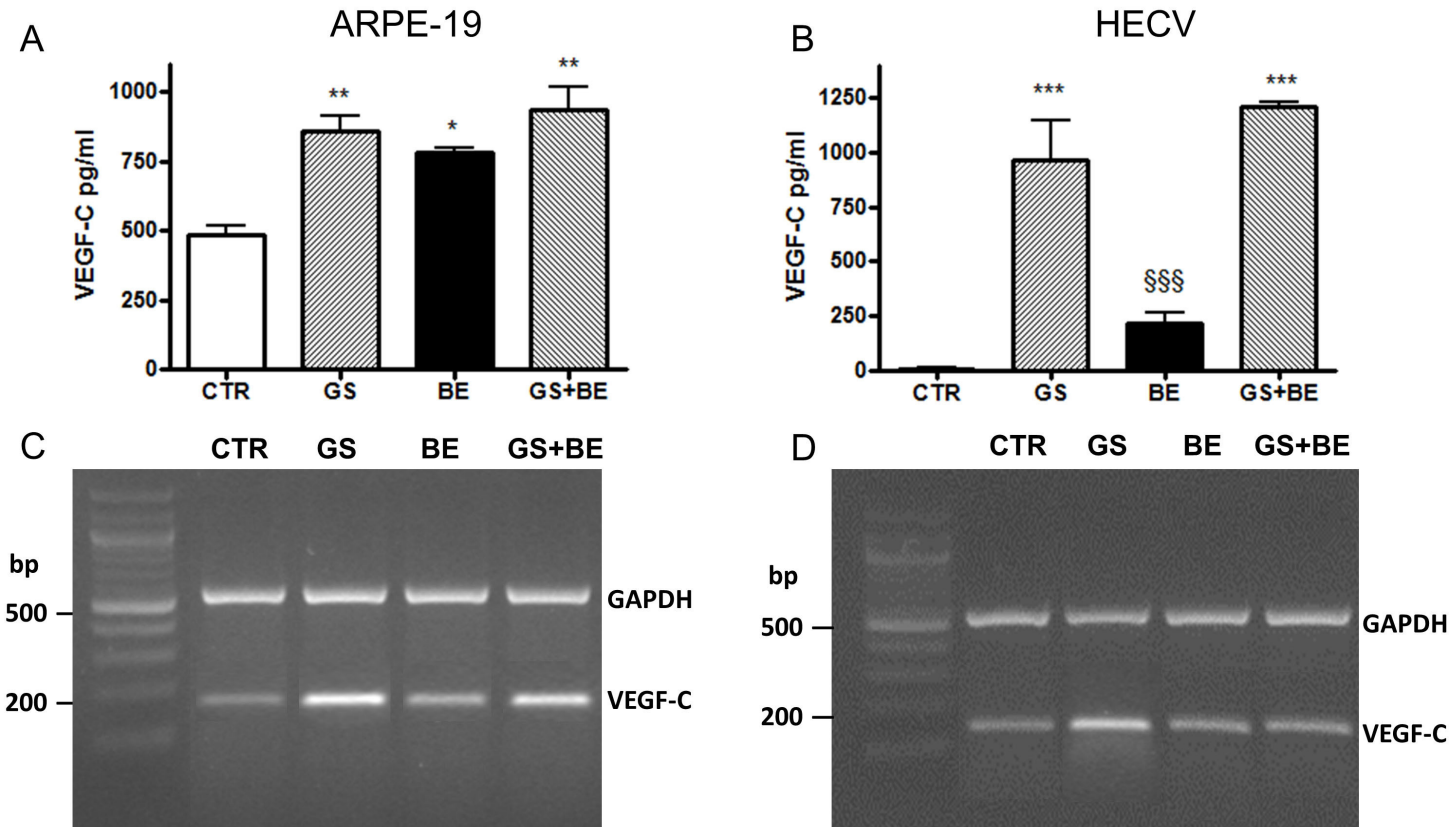
Secretion of vascular endothelial growth factor-C (VEGF-C). Determination of VEGF-C secretion with ELISA (upper panel) and mRNA expression by RT–PCR (lower panel) in ARPE-19 (**A**, **C** and HECV **B**, **D** cells cultured 24 h in standard conditions (CTR) or in the presence of AGEs (GS) with or without addition of bevacizumab (BE). *p<0.05, **p<0.01 and *** p<0.001 versus CTR; §§§p<0.001 versus GS+BE.*p<0.05, **p<0.01 and *** p<0.001 versus CTR; ^§§§^p<0.001 versus GS+BE.

Since VEGF-C secretion may be positively regulated by VEGF-A signal [11], we blocked secreted VEGF-A by adding BE to the culture medium. The results showed that BE markedly increased VEGF-C secretion (Figure 4A,B), but did not affect AGEs-induced VEGF-C secretion.

To determine whether the increase in VEGF-C secretion induced by BE was coupled to increments in VEGF-C expression, RT–PCR was performed. The results showed that BE upregulated VEGF-C mRNA expression in ARPE-19 cells, but not in HECV (Figure 4C,D).

## Discussion

In this work, we show for the first time that AGEs increase VEGF-C secretion in RPE and endothelial cells. The RPE is known to produce and secrete various growth factors that are essential for maintaining the structural integrity of the retina and choriocapillaris. Among them, VEGF-A is secreted in low concentration by the RPE of the healthy eye. Overproduction of VEGF-A plays an essential role in the development of retinal neovascularization, and has been causally linked to many changes that characterize diabetic retinopathy, such as increased capillary permeability and neovascularization [3,14,16,28]. AGEs are important mediators of vascular diabetic complications, including retinopathy [13,14]. The proangiogenic effect of AGEs in retinal neovascularization has been associated with their ability to increase the expression and secretion of VEGF-A [16,25]. Recently, understanding the mechanisms underlying VEGF-C secretion has become critically important due to the increased evidence that this secretion contributes to retinal angiogenesis. Indeed, VEGF-C sustains retinal neovascularization, by potentiating the angiogenic action of VEGF-A and by preventing retinal endothelial cell apoptosis [11,12].

Since hyperglycemia and oxidative stress are associated with glycation [29,30], we hypothesized that, as observed for VEGF-A, AGEs might play a role in regulating VEGF-C secretion. Our results showed that AGEs are able to upregulate VEGF-C expression and secretion in ARPE-19 and endothelial cells.

In our experimental models, downregulation of RAGE expression results in decreased levels of constitutive VEGF-A in RPE and in endothelial cells, suggesting the presence of a regulatory role of RAGE in VEGF-A release. In addition, in RPE cells, RAGE downregulation leads to enhanced secretion of VEGF-C, suggesting that VEGF-C may compensate for VEGF-A depletion. The rates of RAGE expression seem to be important in regulating AGEs-induced VEGFs secretion. Indeed, when AGEs-induced upregulation of RAGE expression is prevented by siRAGE RNA, AGEs fail to increase VEGF-A release in ARPE-19 cells. On the contrary, AGEs maintain the ability to increase VEGF-C secretion. Our results suggest that the mechanism involved in upregulating VEGF-C secretion by AGEs is different from that responsible for VEGF-A release, and that upregulating RAGE expression is necessary for AGEs-induced VEGF-A secretion.

Since an increment in VEGF-A secretion upregulates VEGF-C release in RPE cells [11], it can be speculated that the increased secretion of VEGF-A induced by AGEs may contribute to the AGEs-mediated increase in VEGF-C release. However, a blockade of secreted VEGF-A with bevacizumab does not affect AGEs-induced VEGF-C secretion, suggesting that VEGF-A does not take part in AGEs-induced VEGF-C release. We also found that bevacizumab upregulates constitutive secretion of VEGF-C in RPE and endothelial cells. The increased VEGF-C secretion induced by bevacizumab may be a compensatory response of the cells due to the loss of the autocrine feedback of VEGF-A. This hypothesis is supported by our findings on the secretion of VEGF-A and VEGF-C associated with downregulation of RAGE expression. Our results suggest that in RPE cells the reduction in VEGF-A secretion is counterbalanced by increment in VEGF-C release. Interestingly, in clinical practice more than half of patients do not improve after treatment with monoclonal antibodies against VEGF-A and can be considered nonresponders [31-33]. It has been reported that the major mechanisms of resistance to VEGF-A blockade include signaling by redundant receptors and other forms of VEGFs [34]. Considering that overexpression of VEGF-C has been shown to promote choroidal neoangiogenesis [12], our findings suggest that the observed insensitivity toward anti-VEGF-A therapies may be attributable to the enhanced levels of VEGF-C.

The mechanism through which AGEs increase VEGF-C secretion remains to be fully clarified. Our results show that the effect of AGEs on the production of VEGF-C is independent of RAGE upregulation, and of the effect of secreted VEGF-A, and that the blockade of VEGF-A seems to contribute in enhancing VEGF-C release.

In conclusion, our study demonstrates that AGEs increase the secretion of VEGF-C by RPE and endothelial cells, and that AGEs may take part in the pathologic ocular neovascularization not only by increasing the expression and secretion of VEGF-A but also by increasing VEGF-C secretion. Since VEGF-C enhances the VEGF-A action [11], prevention of VEGF-C secretion should be considered in designing new therapies for diabetic retinopathy, or in potentiating the currently available experimental anti-VEGF-A treatment [35]. Although this study reveals interesting results, there are some limitations concerning the clinical applicability of these findings because the study was conducted in vitro using cell line models, which represent a simplified system compared to the retina.
